# Talking about alcohol to patients (TAP) study: Assessing patient-provider communication of alcohol's breast cancer risks

**DOI:** 10.1016/j.pmedr.2026.103592

**Published:** 2026-08-01

**Authors:** Sumayah Abed, Salma Aly, Gracie Trulove, Vinitha Ganesan, Priscilla Martinez, Ritu Aneja

**Affiliations:** a Department of Family and Community Medicine, The University of Alabama at Birmingham, Birmingham, AL, United States of America; b The University of Alabama at Birmingham Heersink School of Medicine, Birmingham, AL, United States of America; c Department of Nutrition Sciences, The University of Alabama at Birmingham, Birmingham, AL, United States of America; d Alcohol Research Group, Public Health Institute, Emeryville, CA, United States of America

**Keywords:** Alcohol, Breast cancer, Primary care, Provider counseling, cancer prevention, Health communication

## Abstract

**Background:**

Primary care providers are well-positioned to educate patients about alcohol-related health risks, such as breast cancer, but little is known about their awareness of the alcohol-breast cancer (ABC) link or their counseling practices. This study aimed to assess the level of awareness of alcohol as a breast cancer risk factor among providers; to assess the prevalence of provider counseling discussions with their patients about the alcohol-breast cancer link; and identify predictors of level of awareness and counseling discussion about the alcohol-breast cancer link.

**Methods:**

We conducted a cross-sectional survey of primary care providers in Alabama between September and December 2023. A 3-min online questionnaire assessed provider knowledge of the ABC link, counseling practices, and perceived barriers to patient education. Descriptive statistics, bivariate analyses, and logistic regression were used to identify predictors of awareness and counseling behavior.

**Results:**

Among 123 respondents, only 19.5% reported counseling patients about the ABC link. Awareness was significantly higher among counseling providers (95.8%) compared to non-counseling providers (41.7%, *p* < 0.001). Older age and female gender were associated with greater awareness (OR = 1.08 per year, *p* = 0.001; OR = 5.26 for females, *p* = 0.003). Counseling was more likely among providers who were female (OR = 5.26, *p* = 0.041), did not engage in binge drinking (OR = 12.5, *p* = 0.045), and had higher patient loads (OR = 1.02 per patient, *p* = 0.019). The most cited barrier to counseling was lack of awareness (49.4%), while increased provider education was the most frequently proposed solution (45.3%).

**Conclusions:**

Despite the critical role of providers in preventive health, most providers do not counsel patients about the ABC link. Awareness is a key driver of counseling but not sufficient on its own. Targeted educational interventions are needed to enhance provider engagement in alcohol-related cancer prevention.

## Highlights

• Few primary care providers counsel on alcohol and breast cancer risk.
• Awareness of the alcohol–breast cancer link drives counseling uptake.
• Female sex and older age were linked to higher awareness.
• Counseling was more common among females and non-binge drinkers.
• Lack of awareness was the leading barrier to patient counseling.
• Targeted education may improve alcohol-related cancer prevention.

## 1 Introduction

Breast cancer is the second most common cancer among women in the United States, placing substantial health and emotional burdens on individuals, families, and communities. In 2024, over 264,000 new cases of breast cancer were reported among women in the U.S., indicating a concerning rise in incidence. (American Cancer Society, 2022) In a recent statement, the U.S. Surgeon General emphasized the link between alcohol consumption and an increased risk of multiple cancers, including breast cancer, highlighting alcohol as a preventable cause of cancer. (Health, U.S.D.o. and S. Human, 2024) Modifying lifestyle behaviors presents a significant opportunity to reduce breast cancer incidence. Notably, alcohol use is a well-documented risk factor, with studies demonstrating a dose-dependent relationship between alcohol consumption and breast cancer risk. (Bagnardi et al., 2015; Scoccianti et al., 2014; Shield et al., 2016)

Awareness of ABC link is low in the United States. In a recent study, less than 25% of respondents among U.S. women aged 18 years and older were aware of this link, with awareness being particularly low among Black women. (Swahn et al., 2024) Efforts to communicate the link between alcohol use and breast cancer to the general population of women have been limited to date. Mass media communication efforts about the ABC link have occurred minimally outside of the U.S., with notable campaigns in Denmark and Australia. (Christensen et al., 2019; Dixon et al., 2015) In the U.S., only one significant social media campaign, the “Drink Less for Your Breasts” campaign, has aimed to raise awareness about the issue. (National Breast Cancer, F., 2024)

Recent data from the Alcohol and Breast Cancer Link Awareness (ABLE) survey suggest that primary care providers could play a key role in helping disseminate not only general health advice but the specific message that alcohol use is a modifiable risk factor for breast cancer development. (Swahn et al., 2024) In the ABLE survey, half of women reported that their primary doctor would be the first place they would go for information about breast cancer specifically, ranking highest among all other options. (Swahn et al., 2024) Importantly, 40% of the women in the survey reported that their primary care doctor is a trusted source of information about alcohol use and breast cancer (Swahn et al., 2024). Trust in the source of information is key for a message to be believed, and primary care settings thus present an opportunity to address the low awareness of alcohol use as a modifiable risk factor of a breast cancer risk factor. Primary care providers play a crucial role in discussing alcohol use and its health effects with patients, and as a source of prevention guidance for breast cancer (Colditz and Bohlke, 2014). However, the evidence is equivocal on the prevalence of provider discussions with patients about alcohol use and its health consequences (Tan et al., 2018; Denny et al., 2016), and, to the best of our knowledge, there are no studies on the prevalence of primary care provider discussions about alcohol use and breast cancer specifically in the US. This study aims to fill these gaps by 1) assessing primary care providers' awareness of alcohol as modifiable risk factor of a breast cancer measured, 2) estimating the prevalence of provider counseling discussions with female patients about ABC link measured, 3) determining correlates of physicians' level of awareness and provision of counseling discussions about the ABC link, and 4) identifying obstacles to offering counseling about the ABC link and proposed solutions.

## 2 Methods

### 2.1 Study design and population

It's a cross-sectional study. A brief (3-min) Qualtrics survey was developed and distributed to primary care providers including physicians, nurse practitioners (NPs), and physician assistants (PAs) specializing in Family Medicine, Internal Medicine, and Med-Peds. The survey was distributed via a project-specific email account to primary care providers within the academic institution and those listed in the State Academy of Family Physicians directory. Survey distribution began in September 2023. No incentives were offered for participation. Reminder emails were sent every two weeks for two months to non-respondents. Data collection concluded in December 2023. The target sample size was 500 respondents.

### 2.2 Measures

The survey collected information on provider demographics (age, sex, race, and ethnicity), professional background (specialty, clinical setting, years in practice, and patient load), awareness of alcohol as a breast cancer risk factor, counseling practices related to alcohol and breast cancer risk, experience with breast cancer care, and personal and family history of alcohol use and alcohol-related disorders.

Patient load was defined as the number of patients seen by each provider.^ (Bodenheimer and Sinsky, 2014) Awareness of the alcohol–breast cancer association was assessed by asking providers whether they believed alcohol consumption increases breast cancer risk. Counseling practices were assessed by asking providers whether they discussed the alcohol–breast cancer association with female patients.

Information on alcohol use included current alcohol consumption and binge drinking. Binge drinking was defined as consuming five or more drinks on a single occasion for males and four or more drinks on a single occasion for females.^ (Naimi et al., 2003) Family history variables included family history of breast cancer and family history of alcohol-related disorders. The study was approved IRB- 300011138.

### 2.3 Statistical analysis

For continuous variables, normality was assessed using the Shapiro-Wilk test and visual inspection of histograms. The variables were summarized using means and standard deviations (SD), medians and ranges. Categorical variables as outcome variables were described using frequencies and percentages. For comparing groups, Mann-Whitney *U* test was employed for non-normally distributed continuous variables. Chi-square test was used for categorical variables with expected cell frequencies ≥5, while Monte Carlo simulation and Fisher Exact tests were applied for categorical variables with expected cell frequencies <5 to ensure reliable *p*-values.

Logistic regression models were constructed to identify predictors of providers' awareness about the alcohol-breast cancer risk and their counseling practices. Given the relatively small sample size relative to the number of predictors in the model, Firth logistic regression was employed to enhance the stability of the estimates and reduce bias associated with maximum likelihood estimation (Heinze and Schemper, 2002) Covariates were initially selected based on a review of the literature and their statistical significance in bivariate analyses. Models were adjusted for age, gender, ethnicity, race, current alcohol consumption, binge drinking, and practice characteristics; Model 2 additionally included family history (breast cancer and alcohol-related disorders), patient load, and providers' specialty. To further ensure the robustness of the models, multicollinearity among the independent variables (both continuous and categorical) was evaluated using the variance inflation factor (VIF), and only variables with a VIF ≤ 5 were retained in the final models (O'brien, 2007).

Results of models were presented as odds ratios (OR) with corresponding 95% confidence intervals (CI). Reference categories were explicitly defined for categorical variables in the models. Statistical significance was set at *p* ≤ 0.05. All analyses were conducted using R statistical software (R Foundation for Statistical Computing, Vienna, Austria).

## 3 Results

A total of 1089 providers were identified and received the email invitation and survey link. 123 providers initiated the survey and 108 completed all items, yielding a final response rate of 11.3%.

The study population (*N* = 123) had a mean age of 45.6 years (SD = 12.3) and an equal gender distribution. Most participants were non-Hispanic and White, with family medicine being the most common specialty, followed by internal medicine. Providers primarily practiced in community or academic/residency settings (Table 1).

**Table 1:** Demographic, clinical, and behavioral characteristics associated with counseling about the alcohol–breast cancer link among primary care providers in Alabama, United States, September–December 2023.

Characteristics n (%)	Provides counseling on ABC link		Total *n* = 108	*p*-value
	No n = 84	Yes n = 24		
**Demographic characteristics**				
Age⁎				0.03⁎⁎
Mean (SD)	43.7 (11.5)	51.2 (12.2)	45.6 (12.3)	
Gender				0.82
Female	43 (51.2)	11 (45.8)	54 (50.0)	
Male	41 (48.8)	13 (54.2)	54 (50.0)	
Ethnicity				0.98
Non-Hispanic	82 (97.6)	24 (100)	106 (98.2)	
Hispanic	2 (2.4)	0 (0)	2 (1.8)	
Race				>0.99
White	69 (82.1)	19 (79.2)	88 (81.5)	
African American	5 (6.0)	2 (8.3)	7 (6.5)	
Other	10 (11.9)	3 (12.5)	13 (12.0)	
**Clinical characteristics**				
Work experience [years]				<0.01
Mean (SD)	10.1 (10.8)	18.8 (12.4)	10.8 (11.8)	
Clinical specialty				0.42
Family medicine	65 (77.4)	19 (79.2)	84 (77.8)	
Internal medicine	14 (16.7)	2 (8.3)	16 (14.8)	
Other	5 (6.0)	3 (12.5)	8 (7.4)	
Clinical setting				0.61
Community practice	43 (51.2)	15 (62.5)	58 (53.7)	
Residency/academic practice	30 (35.7)	6 (25.0)	36 (33.3)	
Hospital based practice	11 (13.1)	3 (12.5)	14 (13.0)	
**Awareness about increased risk of developing breast cancer from alcohol**				
Don't know	24 (28.6)	1 (4.2)	25 (23.1)	<0.01⁎⁎
No	25 (29.8)	0 (0)	25 (23.1)	
Yes	35 (41.7)	23 (95.8)	58 (53.8)	
**Family history**				
Breast cancer⁎				0.98
No	44 (52.4)	12 (50.0)	56 (52.3)	
Yes	39 (46.4)	12 (50.0)	51 (47.7)	
Alcohol related disorders⁎				0.32
No	39 (46.4)	8 (33.3)	47 (44.3)	
Yes	43 (51.2)	16 (66.7)	59 (55.7)	
**Alcohol consumption within last 30 days**				
Alcohol consumption⁎				> 0.99
Yes	56 (66.7)	16 (66.7)	72 (68.6)	
No	25 (29.8)	8 (33.3)	33 (31.4)	
If yes, is it binge drinking (*n* = 72) ⁎				0.06
Yes	11(19.6)	0 (0.0)	11 (15.3)	
No	44 (78.6)	16 (100)	60 (83.3)	
**Drinking behavior (Among providers who reported drinking alcohol, n = 72)**				
	*n* = 56	*n* = 16	n = 72	
Number of days per month they consume ≥1 alcoholic beverages				0.44
Mean (SD)	7.98 (8.15)	7.56 (9.20)	7.89 (8.33)	
Number of drinks consumed on average				0.63
Mean (SD)	1.43 (0.683)	1.44 (0.512)	1.43 (0.645)	
Number of times in the past 30 days they consumed ≥1 drinks in an occasion				–
Mean (SD)	0.345 (0.844)	0 (0)	0.268 (0.755)	
Largest number of drinks consumed on any occasion				0.43
Mean (SD)	2.44 (1.48)	2.00 (0.96)	2.34 (1.38)	

Abbreviation: ABC link; Alcohol–breast cancer link.

⁎ Variables that have missing data reported.

⁎⁎ Test of statistical significance: *p*-value ≤0.05.

Table 1 presents demographic, clinical, and behavioral characteristics stratified by whether providers counseled patients about the alcohol–breast cancer (ABC) link. Providers who reported counseling patients (*n* = 24) were significantly older than those who did not (*n* = 84) and had longer work experience (both *p* < 0.05; Table 1).

Awareness of the ABC link differed significantly by counseling status. Providers who counseled patients were substantially more likely to endorse alcohol use as a breast cancer risk factor, whereas uncertainty regarding the association was more common among non-counseling providers (*p* < 0.1; Table 1).

Family history of breast cancer and alcohol-related disorders was common in the overall sample and did not differ by counseling status (Table 1).

Alcohol consumption patterns were similar between groups, with comparable proportions reporting recent alcohol use and similar drinking frequency and quantity (Table 1). Although none of the counseling providers reported binge drinking, a higher proportion of non-counseling providers did, representing a marginally significant difference (*p* = 0.06; Table 1).

Table 2 presents the results of a logistic regression analysis examining factors associated with healthcare providers' awareness of the alcohol-breast cancer risk relationship. The model identified two significant predictors of provider awareness. Age emerged as a significant predictor, with each additional year of age increasing the odds of being aware of the alcohol-breast cancer association by 8% (OR = 1.08, 95% CI, 1.03, 1.14). Gender was also a strong predictor, with male providers having significantly lower odds of being aware of the alcohol-breast cancer association compared to female providers (OR = 0.19, 95% CI, 0.05, 0.59). This represents 81% lower odds of awareness among male providers relative to their female counterparts. Other demographic factors showed no significant associations with awareness.

**Table 2:** Multivariable logistic regression analysis of factors associated with awareness of the alcohol–breast cancer association among primary care providers in Alabama, United States, September–December 2023.

Predictors in the model	Odds Ratio	Confidence Interval 95%	*p*-value
Age in years	1.08	1.03, 1.14	<0.01⁎
Gender⁎⁎: Male	0.19	0.05, 0.59	<0.01⁎
Ethnicity⁎⁎: Hispanic	0.24	0.01, 3.57	0.28
Race⁎⁎: White	1.73	0.33, 9.47	0.51
Race⁎⁎: African American	0.96	0.08, 11.18	0.98
Current alcohol consumption	1.22	0.38, 3.91	0.73
Binge drinking⁎⁎⁎ within last 30 days	1.21	0.23, 6.56	0.82
Setting⁎⁎: Hospital-based	0.70	0.13, 3.71	0.67
Setting⁎⁎: Residency/academic	0.70	0.22, 2.22	0.54

⁎ Test of statistical significance: *p*-value ≤0.05.

⁎⁎ Female, non-Hispanic, other races, and community practice settings are reference categories for gender, ethnicity, race, and setting, respectively.

⁎⁎⁎ Binge drinking: For males, drinking ≥5 drinks per occasion and for females, ≥4 drinks per occasion.

Notably, providers' personal alcohol consumption patterns, including current alcohol consumption (OR = 1.22, 95% CI, 0.38, 3.91) and binge drinking (OR = 1.21, 95% CI, 0.23, 6.56), did not significantly predict awareness of the alcohol-breast cancer relationship.

Providers in residency/academic practices showed lower odds of awareness compared to those in community practice settings (OR = 0.70, 95% CI, 0.22, 2.22), but this difference was not statistically significant. Similarly, hospital-based practitioners showed lower odds of awareness compared to community practitioners, though this difference also did not reach statistical significance (OR = 0.70, 95% CI, 0.13, 3.71).

Table 3 shows the multivariable logistic regression examining factors associated with providers' counseling about alcohol and breast cancer risk. Age (per year) was positively associated with counseling (OR = 1.05; 95% CI, 0.99, 1.13) but did not reach statistical significance. Male providers had substantially lower odds of counseling compared with female providers (OR = 0.19; 95% CI, 0.02, 0.94), corresponding to an approximately 81% lower odds of counseling. Neither White nor African American race was significantly associated with counseling (see Table 3 for estimates).

**Table 3:** Multivariable logistic regression analysis of factors associated with counseling about the alcohol–breast cancer association among primary care providers in Alabama, United States, September–December 2023.

Predictors in the model	Odds Ratio	Confidence Interval 95%	*p*-value
Age in years	1.05	0.99, 1.13	0.13
Gender⁎⁎: Male	0.19	0.02, 0.94	0.04⁎
Race⁎⁎: White	0.97	0.15, 7.38	0.98
Race⁎⁎: African American	0.72	0.04, 9.48	0.80
Current alcohol consumption	1.58	0.45, 6.33	0.48
Binge drinking within last 30 days	0.08	0.001, 0.96	0.05⁎
Family history of breast cancer	0.73	0.21, 2.33	0.59
Family history of alcohol related disorders	1.70	0.50, 6.09	0.39
Patient load	1.02	1.001, 1.04	0.02⁎
Specialty⁎⁎: Family Medicine	0.30	0.02. 2.67	0.28
Specialty⁎⁎: Internal Medicine	11.13	0.01, 1.95	0.15

⁎ Test of statistical significance: *p*-value ≤0.05.

⁎⁎ Female, other races, absence of a family history of both breast cancer, alcohol-related disorders, and other specialties are reference categories for gender, ethnicity, race, family history of breast cancer, alcohol-related disorders and clinical specialties, respectively.

Current (non-binge) alcohol consumption was not associated with counseling (OR = 1.58; 95% CI, 0.45, 6.33). Binge drinking within the prior 30 days was strongly inversely associated with counseling (OR = 0.08; 95% CI, 0.001, 0.96), corresponding to roughly 92% lower odds of counseling among providers who reported binge drinking.

Family history of breast cancer and family history of alcohol-related disorders were not significant predictors. Patient load was positively associated with counseling, with each additional patient associated with a small but statistically significant increase in the odds of counseling (OR = 1.02; 95% CI, 1.001, 1.04).

Specialty was not significantly associated with counseling; internal medicine providers showed a non-significant trend toward higher odds of counseling (OR = 11.13, 95% CI, 0.01, 1.95). (Table 3).

Table 4 summarizes the primary barriers and proposed solutions related to alcohol–breast cancer risk counseling. The most commonly reported barrier was limited awareness of the alcohol–breast cancer link, followed by perceptions of minimal or no risk and uncertainty regarding specific details. Time constraints were cited less frequently (Table 4). With respect to proposed solutions, providers most often endorsed increased provider education, followed by allocating more time for patient counseling and improving access to patient education materials. Fewer respondents suggested electronic health record decision support or delegating counseling to other members of the healthcare team (Table 4).

**Table 4:** Reported barriers and proposed solutions for counseling about alcohol-related breast cancer risk among primary care providers in Alabama, United States, September–December 2023.

Obstacles	*n* = 83
Not aware about breast cancer risk	41 (49.4)
Think that there is no risk	13 (15.7)
Aware about risk but not sure from details	12 (14.5)
Don't have time	7 (8.4)
Forgot to counsel	5 (6.0)
Aware about risk but didn't receive training on counseling	1 (1.2)
Other causes⁎	4 (4.8)
**Solutions**	*n* = 106
More provider education about risk	48 (45.3)
More time with patients	25 (23.6)
Patient education resources	17 (16.0)
EHR decision support	10 (9.4)
Nurse/Physician Assistant/Medical assistant provide counseling on ABC link	4 (3.8)
Others⁎⁎	2 (1.9)

Abbreviation: ABC link: Alcohol–breast cancer link; EHR: Electronic Health Record.

⁎ Includes: this risk doesn't cross his mind, doesn't see many patients in primary care, most heavy drinkers are men, or talking about increased risk to diseases in general.

⁎⁎ Includes: not a primary provides, all of the suggestions written will help.

## 4 Discussion

This study assessed primary care providers' awareness of ABC link, estimated the prevalence of provider counseling on this topic, determined correlates of awareness and counseling behavior, and identified perceived barriers and proposed solutions to improve counseling practices. Our findings highlight significant gaps in both provider awareness and counseling practices related to the ABC link, with nearly 80% of providers reporting they do not counsel female patients about alcohol's breast cancer risks. Many studies have shown that primary care providers offer cancer prevention counseling at relatively low rates in their practices. (Naimi et al., 2003; Hipp et al., 2022; Mańczuk et al., 2023; McKnight-Eily et al., 2017; Simapivapan et al., 2018)

Our finding that only 58% of providers were aware of the ABC link is concerning given the robust scientific evidence establishing this relationship. (Bagnardi et al., 2015; Scoccianti et al., 2014; Shield et al., 2016) This awareness level is notably lower than what might be expected among healthcare professionals, particularly given that alcohol's carcinogenic effects have been recognized by the International Agency for Research on Cancer since 1988. The gap becomes even more striking when considering that breast cancer is the most common cancer among women (American Cancer Society, 2022), and alcohol represents one of the few modifiable risk factors for this disease. (Scoccianti et al., 2014)

Several provider characteristics were associated with awareness and counseling. Female sex and older age emerged as strong predictors of greater awareness, whereas female gender, absence of binge-drinking, and higher patient volume were all positively associated with counseling behavior. This is counterintuitive, as one might expect time constraints to limit counseling opportunities. Instead, it may reflect increased clinical exposure, greater experience, or more efficient workflows among high-volume providers. The disparity between awareness levels among counseling providers (95.8%) versus non-counseling providers (41.7%) suggests that awareness is indeed a critical prerequisite for counseling behavior. However, the fact that over 40% of non-counseling providers reported awareness of the ABC link but still did not provide counseling indicates that awareness alone is insufficient to motivate counseling behavior. This suggests additional barriers exist beyond simple knowledge deficits.

Our findings align with national trends showing persistent gaps between alcohol screening and effective counseling. McKnight-Eily et al. found that although 81% of adults were screened for alcohol use, only 20% of binge drinkers were advised to reduce intake. (McKnight-Eily et al., 2017) Extending this work, our study focuses specifically on ABC link and reveals even greater deficits in this area. Prior studies attribute underuse of alcohol counseling in cancer prevention to provider discomfort, limited training, time constraints, and uncertainty about evidence or patient reactions. (Simapivapan et al., 2018; Arem et al., 2021; McKnight-Eily, 2020; Peterson et al., 2016) These barriers appear especially pronounced for the ABC link, likely due to its more recent recognition compared with established risk factors such as tobacco. (Arem et al., 2021) Consistent with Mańczuk et al., our findings underscore the underutilized role of primary care in cancer prevention and highlight the need for education and system-level supports, including EHR prompts and team-based care, to strengthen preventive counseling. (Mańczuk et al., 2023). Counseling regarding alcohol use often competes with many other important preventive health topics, including smoking cessation, nutrition, weight management, and physical activity.

Future research should target larger sample sizes, including nationally representative samples, to understand both national prevalences of awareness of the ABC link and counseling for it among providers, while also examining potential regional variations in provider knowledge and practice patterns. Longitudinal studies could examine how awareness and counseling practices change over time, particularly following educational interventions. Qualitative research could provide deeper insights into provider decision-making processes, specific concerns about discussing alcohol and breast cancer with patients, and preferences for educational content and delivery methods. Studies examining patient perspectives on receiving information about alcohol and breast cancer risk from their providers would complement this provider-focused research and inform the development of patient-centered communication approaches.

Several limitations affect the interpretation of our findings. First, the small sample size (*N* = 123), low response rate (11.3%) and regional concentration in Alabama limit generalizability to other geographic areas and healthcare systems. The response rate, while typical for physician surveys, raises questions about potential selection bias. Providers who chose to participate may differ systematically from non-responders in their awareness levels, counseling practices, or interest in alcohol-related health issues. Second, the survey was intentionally designed to be very brief to encourage participation, and because no incentive was provided, but this limited the evaluation of other factors, such as contextual or other individual-level factors, that could impact awareness of the ABC link or the provision of counseling about the ABC link to female patients. Third, self-reported data may be subject to social desirability bias, particularly regarding provider alcohol use and counseling practices. Finally, the cross-sectional design prevents causal inferences about the relationships identified.

## 5 Conclusion

Our findings underscore the need for targeted interventions to improve provider awareness and the provision of patient counseling regarding alcohol use and the increased risk of developing breast cancer. Educational initiatives should focus on bridging knowledge gaps and equipping providers with effective counseling strategies. Such efforts can address the lack of awareness of alcohol's increasing breast cancer risk among both patients and primary care providers. Primary care providers have an important but ultimately limited role. Although this study focuses on primary care providers, which is appropriate within the U.S. healthcare system, where preventive counseling is largely delivered in primary care, we acknowledge that in many countries, gynecologists serve as the primary providers of preventive women's health care. This distinction may influence the generalizability of our findings.

Future research should explore the development and implementation of a structured curriculum to enhance provider competence and confidence in discussing this critical health risk with patients, and in understanding patient attitudes and perceptions of information about alcohol as a breast cancer risk factor received in a primary care setting. Measures at the population level—such as increasing alcohol prices, reducing availability, public health campaigns, warning labels on alcoholic beverages, and other policy interventions—are essential to implement the changes. More studies should evaluate whether alternative approaches may be effective reach the target population. Public health campaigns, women's health clinics, gynecology practices, community outreach initiatives, social media, warning labels on alcoholic beverages, and population-level educational interventions may represent more effective channels for increasing awareness regarding alcohol-related breast cancer risk.

## CRediT authorship contribution statement

**Sumayah Abed:** Writing – review & editing, Writing – original draft, Project administration, Investigation, Formal analysis, Data curation, Conceptualization. **Salma Aly:** Writing – review & editing, Methodology, Formal analysis. **Gracie Trulove:** Writing – review & editing, Writing – original draft, Project administration, Data curation, Conceptualization. **Vinitha Ganesan:** Writing – review & editing, Project administration, Conceptualization. **Priscilla Martinez:** Writing – review & editing, Resources, Formal analysis, Conceptualization. **Ritu Aneja:** Writing – review & editing, Visualization, Supervision, Resources, Conceptualization.

## Informed consent

The study involved secondary analysis of de-identified data and did not require informed consent in accordance with institutional policies and applicable regulations.

## Ethics approval

This study was reviewed and approved by UAB IRB- 300011138.

## Funding

TAP study received a local Faculty Development Grant Award from University of Alabama at Birmingham of $10.000.

## Declaration of competing interest

The authors declare that they have no known competing financial interests or personal relationships that could have appeared to influence the work reported in this paper.

## Data Availability

Data will be made available on request.

## References

[bb0005] American Cancer Society (2022).

[bb0010] Arem H. (2021). Provider discussion about lifestyle by cancer history: a nationally representative survey. Cancer Epidemiol. Biomarkers Prev..

[bb0015] Bagnardi V. (2015). Alcohol consumption and site-specific cancer risk: a comprehensive dose–response meta-analysis. Br. J. Cancer.

[bb0020] Bodenheimer T., Sinsky C. (2014). From triple to quadruple aim: care of the patient requires care of the provider. The Annals of Family Medicine.

[bb0025] Christensen A.S.P. (2019). Can a mass media campaign raise awareness of alcohol as a risk factor for cancer and public support for alcohol related policies?. Prev. Med..

[bb0030] Colditz G.A., Bohlke K. (2014). Priorities for the primary prevention of breast cancer. CA Cancer J. Clin..

[bb0035] Denny C.H. (2016). Self-reported prevalence of alcohol screening among U.S. adults. Am. J. Prev. Med..

[bb0040] Dixon H.G. (2015). Using a mass media campaign to raise women's awareness of the link between alcohol and cancer: cross-sectional pre-intervention and post-intervention evaluation surveys. BMJ Open.

[bb0045] Health, U.S.D.o. and S. Human (2024).

[bb0050] Heinze G., Schemper M. (2002). A solution to the problem of separation in logistic regression. Stat. Med..

[bb0055] Hipp L.E., Hulswit B.B., Milliron K.J. (2022). Clinical tools and counseling considerations for breast cancer risk assessment and evaluation for hereditary cancer risk. Best Pract. Res. Clin. Obstet. Gynaecol..

[bb0060] Mańczuk M. (2023). Actual and potential role of primary care physicians in cancer prevention. Cancers.

[bb0065] McKnight-Eily L.R. (2020). Screening for alcohol use and brief counseling of adults—13 states and the District of Columbia, 2017. MMWR Morb. Mortal. Wkly Rep..

[bb0070] McKnight-Eily L.R. (2017). Alcohol screening and brief intervention: a potential role in cancer prevention for young adults. Am. J. Prev. Med..

[bb0075] Naimi T.S. (2003). Binge drinking among US adults. JAMA.

[bb0080] National Breast Cancer, F (2024).

[bb0085] O'brien R.M. (2007). A caution regarding rules of thumb for variance inflation factors. Qual. Quant..

[bb0090] Peterson E.B. (2016). Impact of provider-patient communication on cancer screening adherence: a systematic review. Prev. Med..

[bb0095] Scoccianti C. (2014). Female breast cancer and alcohol consumption: a review of the literature. Am. J. Prev. Med..

[bb0100] Shield K.D., Soerjomataram I., Rehm J. (2016). Alcohol use and breast cancer: a critical review. Alcohol. Clin. Exp. Res..

[bb0105] Simapivapan P., Hodge A., Boltong A. (2018). Exploring the provision of alcohol advice by clinicians to breast cancer patients. Eur. J. Cancer Care.

[bb0110] Swahn M.H. (2024). Demographic disparities in the limited awareness of alcohol use as a breast cancer risk factor: empirical findings from a cross-sectional study of U.S. women. BMC Public Health.

[bb0115] Tan C.H. (2018). Screening for alcohol misuse: practices among U.S. primary care providers, DocStyles 2016. Am. J. Prev. Med..

